# 实时经支气管超声引导针吸活检在肺癌分期中的应用

**DOI:** 10.3779/j.issn.1009-3419.2010.05.06

**Published:** 2010-05-20

**Authors:** Ignasi GARCIA-OLIVÉ, José SANZ-SANTOS, Felipe ANDREO, Eduard MONSÓ, 娟 南, 燕 丁

**Affiliations:** 1 Respiratory Service, Hospital Universitari Germans Trias i Pujol, Badalona, Catalunya, Spain; 2 Departament de Medicina. Universitat Autònoma de Barcelona; 3 Ciber de Enfermedades Respiratorias-CibeRes, Bunyola, Spain; 4 天津医科大学总医院，天津市肺癌研究所，天津市肺癌转移与肿瘤微环境重点实验室

**Keywords:** EBUS, 线性经支气管超声引导针吸活检, 肺癌分期, 实时经支气管超声引导针吸活检

## Abstract

线性经支气管超声引导针吸活检（endobronchial ultrasound-guided transbronchial needle aspiration, EBUSTBNA）是新引进的技术，它是实时超声下可视淋巴结的针吸活检。尽管有研究显示，其为肺癌纵隔分期的有效方 法，但全世界多数机构并未应用该技术。本报道旨在分享我们应用EBUS-TBNA的经验，并对相关文献做一简要概 述。我们对有关该技术的已有文献进行综述，并特别介绍了我们应用该技术方面的经验。 EBUS-TBNA用以探查肺 癌患者的转移性纵隔淋巴结和/或肺门淋巴结是有效且安全的。在其它病理状态下，其亦为有效的诊断方法。

## 前言

经气管针吸活检（transtracheal needle aspiration, TTNA）和经支气管针吸活检（transbronchial needle aspiration, TBNA）应用于探查肺癌患者纵隔淋巴结转移已超过30年^[1-3]^。许多研究报道，该技术用于探查支气管癌患者纵隔转移的敏感性超过50%，特异性接近100%^[4-8]^。然而，其敏感性有赖于淋巴结部位（对气管隆突下淋巴结和右侧气管旁淋巴结的敏感性较高）和淋巴结大小，对直径大于20 mm的淋巴结尤为敏感^[9, 11]^。由于敏感性不稳定，该技术未得到广泛应用，仅30%操作呼吸内镜的肺科专家使用了该技术^[12-13]^。

在TTNA或TBNA之前，应用径向支气管内超声（endobronchial ultrasound, EBUS）可见纵隔淋巴结和肺门淋巴结，这使得该项技术的敏感性高达80%，因为在穿刺前可见淋巴结部位的径向超声扫描图像^[14, 15]^。

随后，线性EBUS的出现有助于在实时超声可视淋巴结下实施TTNA或TBNA，这使得穿刺更为精准，而且可以在影像学检查正常的纵隔中进行淋巴结取样^[16-18]^。

最近，有两篇综述评估了EBUS-TBNA在探查肺癌患者转移性纵隔淋巴结中的有效性和安全性^[19, 20]^。

## 方法

EBUS的可弯曲支气管镜安置有末梢弯曲的线性探头（图 1），可产生超声图像，并可对纵隔和支气管周围组织进行线性平行扫描。气管镜有一个工作通道，适宜在直接超声可视下实施TBNA。推荐采用局部利多卡因喷雾和静脉注射咪达唑仑实施局部麻醉和镇静^[21, 22]^。将气管镜置于气管或主支气管部位可探查纵隔淋巴结[气管隆突下淋巴结（图 2）、主肺动脉窗淋巴结、右侧气管旁淋巴结（图 3）和左侧气管旁淋巴结（图 4）]和肺门淋巴结（图 5），并可测量其直径。在这一过程中，对于探查到的短轴直径超过3 mm的淋巴结，可以在直接超声可视下采用专门设计的21 G或22 G细胞学穿刺针进行取样^[18]^，并实施快速的现场细胞学检查。首先对N3区域的所有可到达的淋巴结进行取样，随后为N2和N1区域的淋巴结，直至得到恶性肿瘤的阳性结果。所得组织用95%乙醇固定，苏木素-伊红染色，并根据其是取自含有淋巴细胞的正常淋巴结或取自含有肿瘤细胞群的转移性淋巴结以分类。吸取物中含有的组织太少将不能得到令人满意的结果，在这种情况下，我们可以重复操作，最多3次^[23]^。

**1:**
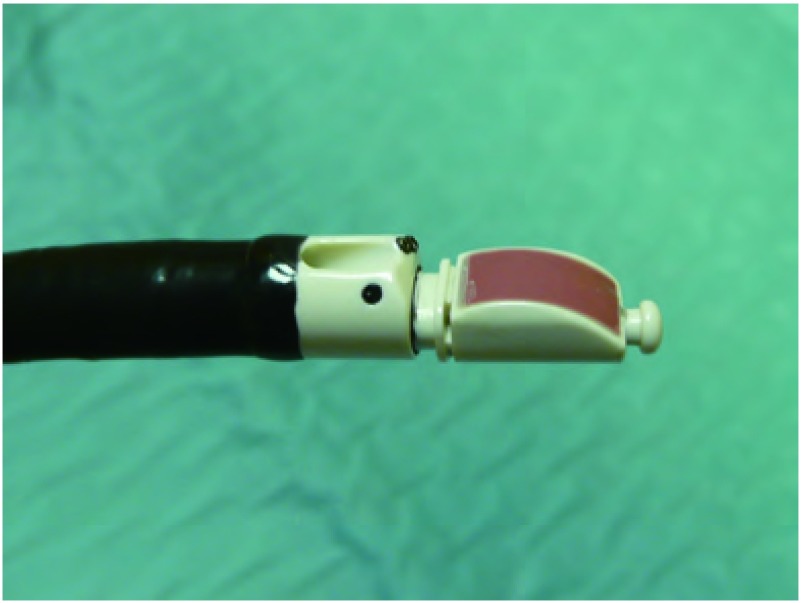
末梢弯曲的线性探头 Distal curved linear probe

**2:**
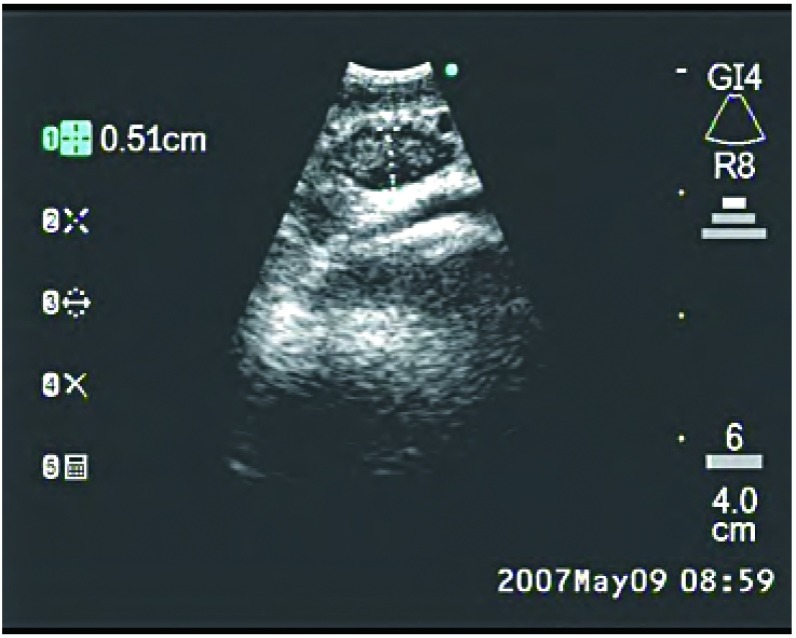
支气管内超声所见气管隆突下淋巴结 Endobronchial ultrasound of subcarinal node

**3:**
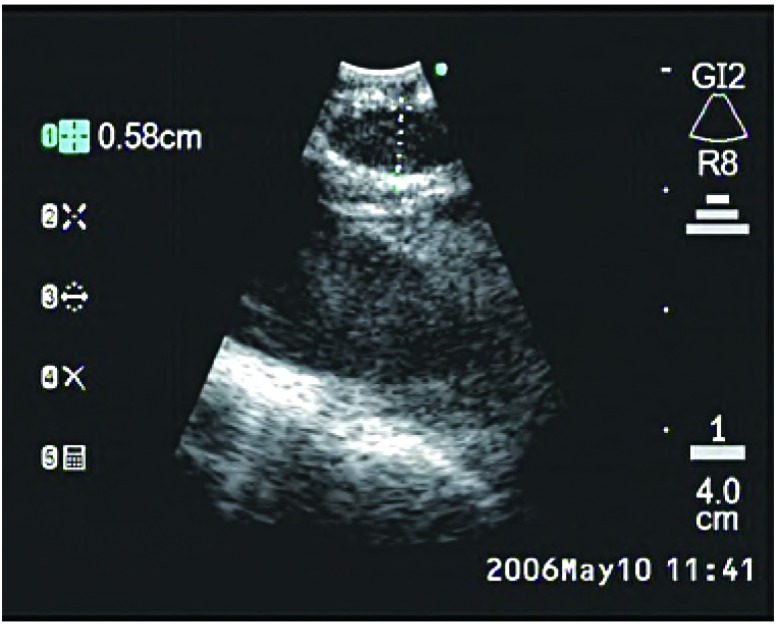
支气管内超声所见右侧气管旁淋巴结 Endobronchial ultrasound of right paratracheal node

**4:**
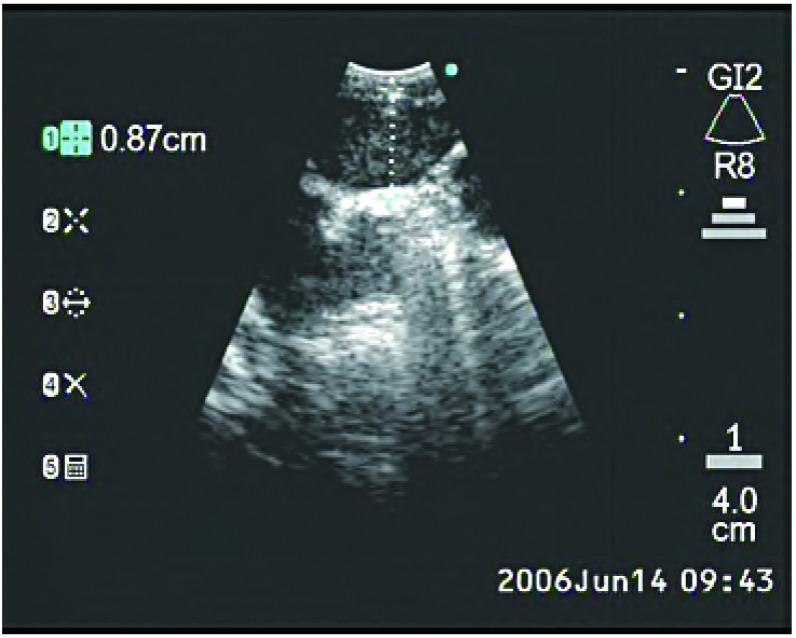
支气管内超声所见左侧气管旁淋巴结 Endobronchial ultrasound of left paratracheal node

**5:**
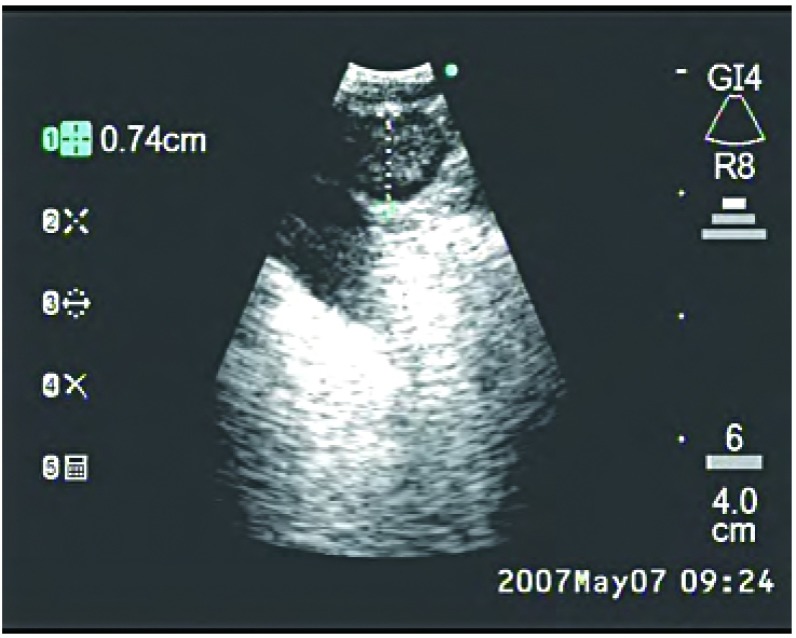
支气管内超声所见肺门淋巴结 Endobronchial ultrasound of hilar node

## 安全性

在本机构所研究的全部患者的操作过程中，以及随后的48 h内，均未出现操作相关的并发症。目前已有之前提及的综述的网络版^[19, 20]^。据其所述，可能的并发症有气胸^[24]^、血氧不足^[25]^、焦虑不安^[26]^、咳嗽^[24, 26]^及穿刺部位出血^[27]^。但是，这些并发症极为罕见。

## EBUS在肺癌分期中的作用

自2003以来，许多关于肺癌分期的研究^[14-18, 24, 25, 27-34]^已经发表。在这些研究中，EBUS-TBNA均呈现出较高的敏感性和特异性。在其中部分研究中，研究者对EBUSTBNA与CT-PET或CT等其它分期方法进行了比较，结果显示，与其它成像技术相比，EBUS-TBNA具有较高的敏感性和特异性^[30]^。

EBUS-TBNA令人感兴趣的另一方面是，它可以从影像技术无法探查到的小于1 cm的淋巴结中取样^[16, 18]^。

EBUS-TBNA的主要局限是观察不到后面的淋巴结（5、7、8和9站）并从其中取样^[20, 30]^。在我们的操作过程中，在特定情形下，EBUS-TBNA可见并可从5站淋巴结中取样。

遗憾的是，未有证据显示纵隔淋巴结的超声图像特征可预测恶性，因此需对所有淋巴结进行取样，尤其是肿大和圆形的淋巴结^[18]^，首先对N3区淋巴结取样，如果此处淋巴结未呈现恶性，则对N2区和N1区淋巴结取样。

我们采用该技术的体验良好。我们发现，在CT扫描呈现正常纵隔的大约30%的患者中，EBUS-TBNA检测肿大淋巴结的结果显示，25%的淋巴结为恶性^[18]^。此外，我们之前的报道指出，超过1/2的患者无需行纵隔镜检查^[18]^，这意味着由于无需住院，外科并发症和费用将减少。

## EBUS可作为诊断方法

尽管大多数研究关注了EBUS在肺癌分期中的应用，但有些研究者认为EBUS-TBNA是诊断淋巴瘤^[35]^、结节病^[36-38]^、肺结核^[38]^或肺癌的有效方法^[38, 39]^。

## 内镜超声和EBUS

据报道，内镜超声（endoscopic ultrasonotraphy, EUS）与EBUS联用是创伤几乎最小的肺癌患者纵隔分期方法，可使某些患者避免进行纵隔镜检查^[28, 33, 40]^。

## 总结

EBUS-TBNA用以探查肺癌患者甚至影像学检查纵隔正常的患者的转移性纵隔淋巴结和/或肺门淋巴结是有效且安全的。对于其它纵隔疾病，其亦为有效的诊断方法。EUS-TBNA与EBUS-TBNA联用几乎可以对整个纵隔进行取样。这将使纵隔镜检查的次数减少。

## Conflict of Interest

I Garcia-Olivé, J Sanz-Santos, F Andreo and E Monsó do not have any financial or personal relationships with other people or organizations that could inappropriately influence their work in the present study.

## Acknowledgments

Our work has been supported by funds from the CIBER de Enfermedades Respiratorias- CibeRes, Fondo de Investigación Sanitaria FIS 070170, Sociedad Española de Neumologíay Cirugía Torácica (SEPAR), Societat Catalana de Pneumologia (SOCAP), Asociación Española de Endoscopia Respiratoria (AEER) and Redes Temáticas de Investigación Cooperativa en Cáncer RTICC RD06/0020/0056. Ciber de Enfermedades Respiratorias - CibeRes is an initiative of Instituto de Salud Carlos Ⅲ.
